# Low-concentration atropine for management of myopia progression: does iris colour matter?

**DOI:** 10.1038/s41433-026-04478-1

**Published:** 2026-04-27

**Authors:** Gareth Lingham, James Loughman, Samantha SY Lee, Michael X. Repka, Eoin Kerin, Alicia Gómez Sánchez, Emmanuel Kobia-Acquah, Ernest Kyei Nkansah, Ian Flitcroft, David A. Mackey

**Affiliations:** 1 Centre for Ophthalmology and Visual Science (incorporating Lions Eye Institute), University of Western Australia, Perth, WA Australia; 2 Centre for Eye Research Ireland, Sustainability and Health Research Hub, Technological University Dublin, Dublin, Ireland; 3 Ocumetra, Dublin, Ireland; 4 Centre for Eye Research Australia, Melbourne, WA Australia; 5 Wilmer Eye Institute, Baltimore, MD USA; 6 Transitions Optical Limited, Tuam, Ireland

**Keywords:** Medical research, Paediatrics

## Abstract

**Background:**

To investigate interactions between iris colour and low-concentration atropine on outcomes relevant to clinical myopia management

**Methods:**

A post-hoc, exploratory meta-analysis of three randomised clinical trials investigating atropine 0.01% or 0.05% eye drops vs placebo: the Western Australian Atropine Treatment of Myopia study, the Myopia Treatment Study and the Myopia Outcome Study of Atropine in Children. Iris colour was graded as brown or not brown (including blue/green/hazel). Change in accommodative amplitude, pupil diameter, spherical equivalent refraction (SER) and axial length were assessed using linear mixed models.

**Results:**

In participants (baseline ages 5–16 years) using atropine 0.01% (*n* = 396), pupil diameter change did not differ by iris colour (*p* ≥ 0.15), while accommodative amplitude decreased more in not brown vs brown iris colour groups at month 12 (adjusted difference = −1.07D, 95% CI: −2.02, −0.12, *p* = 0.03), but not at month 24 (*p* = 0.76). Change in pupil and accommodative outcomes with 0.05% atropine (*n* = 66) did not differ by iris colour (*p* ≥ 0.10). Among not brown iris colour participants, myopia progression was lower in the atropine 0.01% vs placebo group at 24 months (SER: +0.17D, 95% CI: 0.07, 0.26, *p* < 0.001; axial length: −0.09 mm, 95% CI: −0.12, −0.05, *p* < 0.001), but not among brown iris colour participants (SER: −0.08D, 95% CI: −0.20, 0.05; axial length: +0.03 mm, 95% CI: −0.02, 0.07).

**Conclusion:**

Change in pupil diameter and accommodative amplitude with low-concentration atropine largely did not differ between iris colour groups. Among participants using 0.01% atropine eye drops, there was less 24-month myopia progression compared to placebo in participants with blue/green irides, but not with brown irides.

## Introduction

Myopia is an important cause of irreversible vision loss worldwide [1–3] and vision loss attributable to myopia is predicted to rise as the prevalence and severity of myopia increases [4]. Urbanised regions of East and South-East Asia, particularly, have extreme rates of myopia among younger generations, reaching as high as 70–90% among high school and university students [5, 6]. Moreover, myopic macular degeneration, a complication of myopia, is a growing cause of irreversible, moderate-to-severe vision impairment among Chinese adults [7]. Risk of vision loss increases with increasing myopia severity. Therefore, treatments to slow myopia progression and reduce an individual’s final severity of myopic refractive error are essential to prevent myopia complications and related vision loss [8].

Atropine, a muscarinic antagonist, slows myopia progression. The landmark Atropine for the Treatment of Myopia (ATOM) 1 and 2 studies in Singapore showed that atropine 1%, 0.5%, 0.1% and 0.01% eye drops reduce myopia progression in a dose-dependent manner [9, 10]. Due to fewer side effects related to the cycloplegic action of atropine eye drops, and lower rebound myopia progression upon cessation, atropine 0.01% was identified as the optimal concentration [11]. However, the Low-concentration Atropine for Myopia Progression (LAMP) study in Hong Kong later showed that atropine 0.05% has a more favourable efficacy profile, without significantly worsening adverse effects [12, 13]. The applicability of these studies to populations of European descent has been questioned due to potential interactions with iris melanin (which impacts iris colour) and has prompted further randomised controlled trials [14–17].

Atropine is known to reversibly bind to melanin [18, 19] and eyes with less iris pigment demonstrate a higher peak response to topical administration of similar anti-muscarinic agents [20]. Thus, it is hypothesised that individuals with a lighter iris colour will experience greater mydriasis and loss of accommodative function when using low-concentration atropine eye drops. On the other hand, recent clinical trials have shown that atropine 0.01% is well tolerated in populations with mixed eye colours [14–17]. While overall efficacy varied from modest to no effect in these studies, two – the Western Australian Atropine Treatment of Myopia (WA-ATOM) [15] and Myopia Outcome Study of Atropine in Children (MOSAIC) [14] – reported greater treatment effect sizes among participants of White European descent. However, on subgroup analysis, the only statistically significant difference was for axial length change at 24-months in MOSAIC (difference = −0.09 mm, *p* = 0.002 vs difference = +0.03, *p* = 0.62 for White vs non-White ethnicities, respectively). Neither study was powered to detect subgroup differences. The present post-hoc meta-analysis aims to characterise the relationship between iris colour and accommodative amplitude, pupil diameter and myopia progression in response to low-concentration atropine eye drops.

## Methods

This study utilised data from three randomised clinical trials (RCTs) of low-concentration atropine eye drops that included children and adolescents of European descent: the Irish MOSAIC, the Australian WA-ATOM study and publicly available data from the Pediatric Eye Disease Investigators Group’s Myopia Treatment Study (MTS1).

### RCTs of atropine 0.01% vs placebo: MOSAIC, WA-ATOM, MTS1

The methods and primary outcomes for the MOSAIC, WA-ATOM and MTS1 studies have been previously published [14, 15, 17]. All three studies were double-masked placebo-controlled RCTs. In each, participants were randomly assigned at baseline in a 2:1 ratio to atropine 0.01% eye drops or placebo eye drops to be used nightly in both eyes for 24 months, with participants and outcome assessors masked to the treatment assignment.

Eligibility and assessments were broadly similar across the three studies (Supplementary Table 1). However, some important differences include:

- The eligible age range was younger in MTS1 (5–12 years) vs MOSAIC, WA-ATOM (6–16 years).
- Iris colour was classified dichotomously as brown or not brown in MTS1, but on 9-grade scale for MOSAIC and WA-ATOM [21].
- Pupil diameter under mesopic and photopic conditions was only measured in MOSAIC (Aladdin, Topcon, Japan).
- The 6-month visit of MOSAIC was abandoned due to the COVID-19 pandemic and, for risk mitigation, accommodative amplitude was also skipped at month 12.

### Atropine 0.05% vs placebo: MOSAIC phase 2

There were two phases to the MOSAIC study: MOSAIC1, which is described in the previous paragraph and took place from baseline to month 24 and MOSAIC2, an extension of MOSAIC1 which ran from month 24 to month 36 [22]. In MOSAIC2, beginning at the 24-month visit, participants assigned to placebo eye drops in MOSAIC1 were re-assigned to nightly atropine 0.05% eye drops in both eyes, while participants previously assigned to atropine 0.01% in MOSAIC1 were re-randomised to receive either placebo eye drops (washout) or a 12-month tapering regimen of atropine 0.01% eye drops. Participants and examiners remained masked to both MOSAIC1 and MOSAIC2 treatment assignments and examinations remained consistent across the two phases (Supplementary Table 1).

### Power calculations

Power calculations for MOSAIC, WA-ATOM and MTS1 have been published based on their primary outcomes and endpoints.

### Statistical analysis

**Fig. 1:**
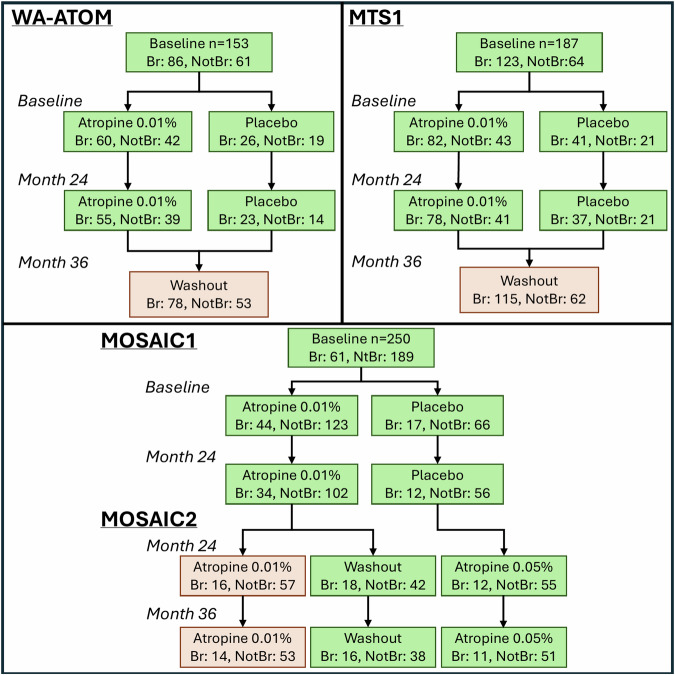
Simplified flowcharts showing relevant treatment assignments and brown iris colour (Br) and not brown iris colour (NotBr) group sample sizes at major visits for each study. Green boxes indicate data that were analysed in the current study whereas data from participants indicated by orange boxes were not included in the current study.

To optimise pooling and comparability of results across studies, this post-hoc, person-level meta-analysis of low-concentration atropine RCTs only included the following (Fig. 1):

- Participants assigned to one drop of atropine 0.01% nightly.
- Participants assigned to one drop of atropine 0.05% nightly.
- Participants assigned to one drop of placebo nightly.

When pooling data, the simplest categorisation schema was used such that iris colour was categorised as brown vs not brown (in line with MTS1) and parent- or participant-reported ethnicity was categorised as either White, Asian, Black, Hispanic or mixed/other (in line with MOSAIC).

Parametric, non-parametric and continuous data were described using mean and standard deviation (SD), median and interquartile range (IQR) and number and percent, respectively. Outcomes for this analysis were change in ocular parameters from baseline (for MOSAIC2, this is the 24-month visit); specifically: spherical equivalent refraction (SER), axial length, accommodative amplitude and pupil diameter under photopic and mesopic conditions.

Comparison of outcomes across treatment and eye colour groups were analysed using linear mixed models (LMMs). To account for within-person and within-study correlations, hierarchical models with random intercept terms for participant (when both eyes were used) nested within study (when more than 1 study was pooled) were constructed. To test whether the effect of low-concentration atropine treatment differed by iris colour group and visit, a three-way interaction between eye colour, treatment and visit was included in the model as well as all lower order interactions (see next sentence) and main effects. If *p* was ≥0.05 for this three-way interaction, a second model was constructed replacing the three-way interaction with three two-way interactions: treatment by iris colour, treatment by visit, iris colour by visit. In this case, the treatment by iris colour interaction tests whether the effect of treatment differed with iris colour group without differing across visits. Type III analysis of variance (ANOVA) tables were used to assess interaction terms and, if significant, pairwise comparisons of estimated marginal means were used to assess between-iris colour group differences and 95% confidence intervals (95% CI) with *p* < 0.05 (two-sided test) considered to be statistically significant. All LMMs were adjusted for age, sex and the baseline value of the outcome (e.g., pupil diameter, axial length) and the residuals evaluated for normality using quantile-quantile plots. No adjustments for multiple testing were made. All analyses were conducted in R (version 4.3.2, R Foundation for Statistical Computing, Vienna, Austria).

## Results

### Enrolment and participant characteristics

Figure 1 shows study enrolment and retention. Briefly, MOSAIC enrolled 250 participants, of whom, 204 (81.6%) completed the 24-month visit and 199 (79.6%) continued to MOSAIC2. Of these 199, 126 (63.3%) participants were using either placebo or nightly atropine 0.05% eye drops. In WA-ATOM and MTS1, 153 and 187 participants were enrolled at baseline, respectively, and 131 (85.6%) and 177 (94.6%) attended the 24-month visits.

Table 1 shows baseline participant characteristics. MTS1 participants were ~1.5 years younger on average than MOSAIC or WA-ATOM participants. The MOSAIC study had a much higher proportion of participants with blue or green eyes (75.6%). Among participants who withdrew, proportions of brown vs not brown iris colours were similar in the combined MOSAIC1, WA-ATOM and MTS1 cohorts at month 24 and the MOSAIC2 cohort at month 36 (Fig. 1).

**Table 1:** Characteristics of participants in the current study from the MOSAIC (MOSAIC1 and MOSAIC2 [extension from month 24 to 36), WA-ATOM and MTS1 studies at their respective baseline visits.

	MOSAIC1	MOSAIC2 (12-month extension of MOSAIC 1)	WA-ATOM	MTS1
*n*	250	126	153	187
Age (years), mean (SD)	11.8 (2.37)	13.7 (2.38)	11.51 (2.69)	10.0 (1.78)
Sex, *n* (%)				
Female	155 (62.0%)	78 (61.9%)	89 (58.2%)	101 (54.0%)
Male	95 (38.0%)	48 (38.1%)	64 (41.8%)	86 (46.0%)
Iris colour, *n* (%)				
Brown	61 (24.4%)	30 (23.8%)	86 (58.5%)	123 (65.8%)
Not brown	189 (75.6%)	96 (76.2%)	61 (41.5%)	64 (34.2%)
Blue	120 (48.0%)	65 (51.6%)	33 (22.4%)	N/A
Green	69 (27.6%)	31 (24.6%)	28 (19.0%)	N/A
Ethnicity, *n* (%)				
White	207 (82.8%)	104 (82.5%)	75 (46.0%)	86 (46.0%)
Asian	22 (8.8%)	10 (7.9%)	66 (43.1%)	26 (13.9%)
Black	4 (1.6%)	3 (2.4%)	1 (0.7%)	34 (18.2%)
Hispanic	1 (0.4%)	0	1 (0.7%)	30 (16.0%)
Mixed/Other	16 (6.4%)	8 (7.1%)	10 (6.5%)	11 (5.9%)
SER (D), median [IQR]	−3.28 [−4.54, −2.06]	−4.00 [−4.87, −2.81]	−3.13 [−4.25, −1.92]	−2.69 [−4.24, −2.50]
Axial length (mm), mean (SD)	24.87 (1.05)	25.21 (1.1)	24.72 (0.81)	24.43 (0.80)
Treatment arm, *n* (%)				
Placebo	83 (33.2%)	60 (47.6%)	49 (32.0%)	62 (33.2%)
0.01% atropine	167 (66.8%)	0	104 (68.0%)	125 (66.8%)
0.05% atropine	0	66 (52.4%)	0	0

MOSAIC2 data only includes participants in the current analysis, that is those assigned to placebo eye drops, atropine 0.01% eye drops nightly or atropine 0.05% eye drops nightly. The not brown iris colour group is comprised of the blue and green/hazel iris colour groups.

For MOSAIC2, the effective baseline visit occurred at month 24. All participants from MOSAIC2 were also in MOSAIC1.

*SER* Spherical equivalent refraction, *SD* standard deviation, *IQR* Interquartile range.

### Accommodative amplitude

**Fig. 2:**
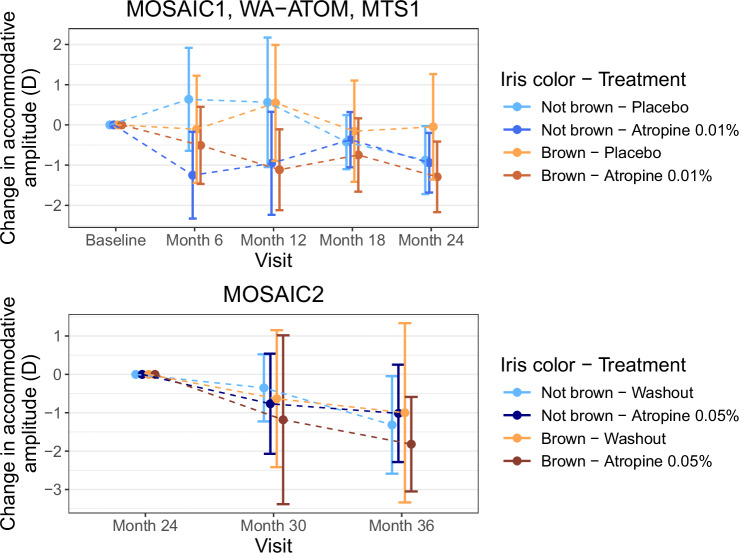
Raw mean change and 95% confidence intervals (error bars) in measured accommodative amplitude (AA) by treatment arm and iris colour group. For pooled WA-ATOM, MOSAIC1 and MTS1 studies (top), the adjusted three-way interaction between treatment, iris colour and visit was significant (*p* = 0.002), meaning the effect of treatment assignment on change in AA differed across iris colour and visit; specifically, AA had decreased more in the not brown iris colour group compared to the brown iris colour group in the atropine 0.01% group at month 12 and in the placebo group at month 24. For MOSAIC2 study (bottom), the adjusted three-way interaction between treatment, iris colour and visit and the two-way interaction between treatment and iris colour were not significant (*p* = 0.53 and *p* = 0.57, respectively) indicating the effect of treatment assignment on AA did not vary across iris colour and visit, or iris colour alone, respectively.

Accommodative amplitude was assessed in the MOSAIC, WA-ATOM and MTS1 trials (Fig. 2, Supplementary Table 2). Accommodative amplitude tended to decline over the full study period. However, over the first 12 months of the WA-ATOM and MTS1 studies (MOSAIC1 data is missing for these visits), there was a small increase in accommodative amplitude in the placebo groups and a small decrease in the atropine 0.01% groups. Compared to the brown iris colour group, the not brown iris colour group had a greater adjusted reduction in accommodative amplitude within the atropine 0.01% group at the 12-month visit (adjusted difference = −1.07D, 95% CI: −2.02, −0.12, *p* = 0.03) as well as within the placebo group at the 24-month visit (adjusted difference = -1.33D, 95% CI: −2.50, −0.16, *p* = 0.03). At all other visits, there were no significant iris colour differences in the placebo or atropine 0.01% eye drop groups. Among MOSAIC2 participants, there was no significant interaction between iris colour, treatment and visit (*p* = 0.53) or iris colour and treatment (*p* = 0.57).

### Static pupillometry

**Fig. 3:**
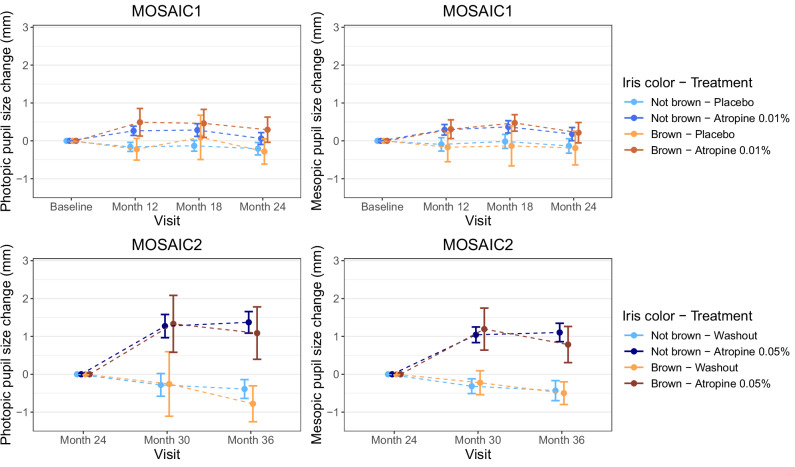
Raw mean change and 95% confidence intervals (error bars) in photopic pupil diameter (left) and mesopic pupil diameter (right) by treatment arm and eye colour group. In MOSAIC2, the extension study, eyes treated with atropine 0.01% in MOSAIC1 (up to 24-month visit) were switched to placebo during MOSAIC2 (month 24 to month 36) explaining the decrease in pupil diameter at the 30- and 36-month visits. Adjusted analyses of three-way interactions between treatment, iris colour and visit, and of two-way interactions between treatment and iris colour were not significant for MOSAIC1 (photopic: *p* = 0.15, *p* = 0.08, respectively; mesopic: *p* = 0.55, *p* = 0.09, respectively) or MOSAIC2 (photopic: *p* = 0.81, *p* = 0.56, respectively; mesopic: *p* = 0.25, *p* = 0.35, respectively).

Pupil diameter under mesopic and photopic luminance was assessed in the MOSAIC study (Fig. 3, Supplementary Table 3). There were dose-dependent increases in mesopic and photopic pupil diameter with low-concentration atropine eyedrops. The effects of atropine 0.01% or atropine 0.05% did not differ significantly by iris colour under mesopic or photopic conditions in MOSAIC1 (*p* all ≥0.08) and MOSAIC2 (*p* all ≥0.25).

### SER and axial length

**Fig. 4:**
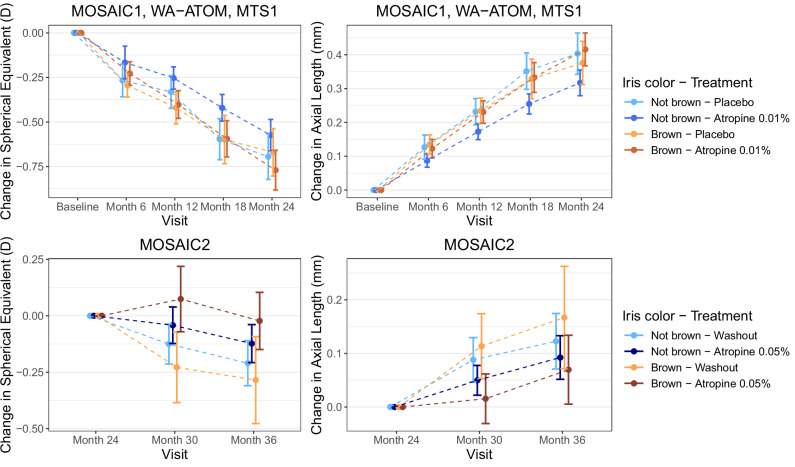
Raw mean change and 95% confidence intervals (error bars) for spherical equivalent and axial length outcomes by treatment arm, visit and iris colour group in the (top) pooled MOSAIC1, WA-ATOM and MTS1 and (bottomt) MOSAIC2 studies. For top plots, three-way interactions between treatment, iris colour and visit were significant (spherical equivalent *p* = 0.04, axial length *p* = 0.01). For bottom plots, both three-way interactions and two-way interactions between treatment and iris colour were not significant (spherical equivalent: *p* = 0.67 and *p* = 0.06, respectively; axial length: *p* = 0.96 and *p* = 0.10, respectively).

The myopia progression outcomes of SER and axial length are shown in Fig. 4 and Supplementary Table 4. In the pooled MOSAIC1, WA-ATOM and MTS1 studies, there were significant interactions between treatment (atropine 0.01% vs placebo), iris colour and visit for SER (*p* = 0.04) and axial length (*p* = 0.01) outcomes. At month 24, within the brown iris colour group, there were no significant atropine 0.01% vs placebo differences for either SER (adjusted difference = −0.08D, 95% CI: −0.20, +0.04, *p* = 0.21) or axial length (adjusted difference = +0.03 mm, 95% CI: −0.02, +0.07, *p* = 0.30). However, among the not brown iris colour group, the atropine 0.01% group had less SER (adjusted difference = +0.17D, 95% CI: +0.06, +0.28, *p* = 0.003) and axial length change (adjusted difference = −0.09 mm, 95% CI: −0.13, −0.05, *p* < 0.001), compared with placebo participants with not brown iris colours. Similar patterns were noted across other visits (Supplementary Table 4). Conversely, at month 36 in MOSAIC2, after 12 months of either atropine 0.05% or washout with placebo, there was no difference in SER or axial length progression by treatment and iris colour group, with all relevant three-way and two-way interactions being non-significant (Fig. 4, Supplementary Table 4).

### Sensitivity analysis

To assess whether ethnicity interacted with iris colour when investigating myopia progression, we conducted a sensitivity analysis of 0.01% atropine vs placebo after restricting to White participants (*n* = 368). Because only 17 of 123 participants assigned to placebo had brown eyes, the not brown and brown iris colour groups using placebo treatments were collapsed and compared directly to participants assigned to atropine 0.01% with brown (*n* = 45) or not brown (*n* = 200) iris colours (Supplementary Fig. 1). There was a significant interaction between the collapsed iris colour/treatment group categories (placebo vs not brown atropine 0.01% vs brown atropine 0.01%) and visit for axial length (*p* = 0.04), but not SER change (*p* = 0.10). At 24 months, the not brown iris colour group using atropine 0.01% at 24 months had significantly less axial length change, compared to the combined placebo group (adjusted difference = −0.09 mm, 95% CI: −0.13, −0.05, *p* < 0.001), but the brown iris colour group using atropine 0.01% did not significantly differ (adjusted difference = −0.03 mm, 95% CI: −0.09, +0.03, *p* = 0.55). Furthermore, we repeated linear mixed models including all participants and adjusting for ethnicity. For both SER and axial length, there were still significant three-way interactions between iris colour, treatment and visit after adjusting for ethnicity when comparing atropine 0.01% vs placebo (SER: *p* = 0.04, axial length: *p* = 0.01), with atropine 0.01% vs placebo group differences similar at 24 months for not brown (SER: adjusted difference = +0.17D, 95% CI: +0.06, +0.28; axial length: adjusted difference = −0.09 mm, 95% CI: −0.05, −0.14) and brown iris colour groups (SER: adjusted difference = −0.08D, 95% CI: −0.21, +0.04; axial length: adjusted difference = +0.03 mm, 95% CI: −0.02, +0.07).

## Discussion

Using data from three RCTs of placebo, atropine 0.01% or atropine 0.05% eye drops, we found that changes in pupil diameter and accommodative amplitude generally did not differ with these low concentrations between the brown and not brown iris colour groups, with some minor exceptions. However, changes in SER and axial length with atropine 0.01% vs placebo assignment did differ by iris colour. Within the not brown iris colour group, participants assigned to atropine 0.01% had less mean axial length change (−0.09 mm) and SER change (+0.17D) compared with placebo at month 24, while there was no difference among the brown iris colour group. In the MOSAIC2 analysis of atropine 0.05% vs washout, we did not detect any iris colour-based differences in SER and axial length change.

### Pupil and accommodation outcomes

We hypothesised that participants with lighter iris colours would experience more pupil dilation and reduction in accommodative amplitude with use of low-concentration atropine eye drop due to lower sequestration of intraocular atropine in the iris melanin. Across multiple studies and treatment arms, our results were largely consistent in showing that this was not the case. The not brown iris colour group had a greater loss of accommodative amplitude with atropine 0.01% use compared to the brown iris colour group at 12 months. At 18 and 24 months, there were no differences in change in accommodative amplitude between iris colour groups, possibly reflecting increasing tolerance to atropine or an increase in statistical certainty with the addition of MOSAIC data for month 18 and 24 timepoints (months 6 and 12 weren’t measured due to COVID-19 pandemic). That we did not find a difference between iris colour groups may be driven by the timescale of the studies. Over months of atropine use, it is likely that atropine that was previously sequestered by melanin was gradually released back into the anterior segment of the eye and the concentration of intraocular atropine became more stable [19, 23]. Our results provide empirical evidence that, with repeated administration of low-concentration atropine eye drops (0.01% and 0.05%), changes in pupil diameter and accommodative amplitude by 24 months are similar between dark and light iris colours.

### SER and axial length outcomes

Compared to placebo groups, we found significantly less myopia progression with atropine 0.01% treatment assignment among the not brown iris colour group; however, there was no such difference among the brown iris colour group. Indeed, the brown iris colour group using atropine 0.01% had approximately the same amount of mean axial length growth over 24 months (0.42 mm) as placebo group participants with either brown (0.38 mm) or not brown (0.40 mm) iris colours. This finding is plausible; the posited site of action of atropine eye drops for myopia control is at the retina, choroid or sclera [24]. Thus, atropine must penetrate the cornea and diffuse posteriorly in the eye to reach its therapeutic site. By virtue of this, the concentration of atropine in the posterior segment of the eye is up to 20-fold lower than in the anterior segment after topical administration [25]. Sequestration of atropine in the anterior segment by iris melanin-binding, which is likely higher in darker irides, could further reduce the concentration of posterior segment atropine, potentially to less than therapeutic levels. Such an explanation is also consistent with our inability to detect a significant iris colour difference with atropine 0.05% vs washout treatment where the higher dose may allow a therapeutic threshold at the retina to be reached. Notably, the smaller sample size in this analysis must be considered. While age is a key factor influencing myopia progression, an additional subgroup analysis by age was not performed because: i) we did not hypothesise that the effect of iris colour would vary with age, and ii) additional subgrouping (within existing subgroups of iris colour) could lead to higher risk of type I and type II errors from uncontrolled confounding and lower sample sizes, respectively.

Our findings, albeit exploratory in nature, suggest that participants with brown iris colours may not respond as well to atropine 0.01% treatment. These results are not consistent with previous RCTs of atropine 0.01% in Asian populations with predominantly brown iris colour, where several studies found a significant treatment benefit from atropine 0.01% eye drops [26–28]. One potential explanation relates to differing atropine eye drop manufacture, with intraocular penetration of atropine impacted by pH and inclusion of preservatives [29]. Of note, MOSAIC and MTS1 used unpreserved 0.01% atropine, while WA-ATOM included a benzalkonium chloride preservative. However, a study of enucleated pig eyes suggests benzalkonium chloride did not improve corneal penetration of atropine [30]. Alternatively, it may be that a larger absolute treatment effect is seen in faster progressing populations such as those found in these Asian RCTs, which also tend to be younger [13, 27, 31]. The LAMP study has shown that higher concentrations of atropine were more effective for myopia control and the MOSAIC2 study has confirmed the tolerability and efficacy of atropine 0.05% in a European population.

### Context

Our findings that accommodation and pupil changes are similar between light and dark iris colour groups are supported by several studies that have investigated this relationship. Two small studies (*n* = 31 participants and *n* = 20 eyes) of short-term atropine 0.01% eye drop use in young adults both reported no difference in pupil dilation between iris colour groups [32, 33], while one additionally reported no difference in symptoms of photophobia [32]. A study of 37 children receiving either 0.01% or 0.05% atropine eye drops in one eye reported no light vs dark iris colour differences in pupil diameter, accommodative amplitude or near vision, but slightly more dark iris colour participants reported visual impairments or reading difficulties (9/19) compared to those with light iris colours (3/18) [34]. Conversely, a retrospective study of 13 children and adolescents using 0.01% or 0.005% atropine eye drops for myopia reported no difference in rates of adverse events. Atropine 0.01% eye drops are reported to be extremely well tolerated with few treatment-related adverse events [14–17]. MOSAIC2 did report a higher prevalence of treatment-related adverse events in participants with blue and green/hazel iris colours, compared to brown iris colours (22% vs 0%, respectively) [22], which might suggest that symptoms of photophobia and blurred near vision do not necessarily correlate with objective measurements of pupil diameter and accommodative amplitude.

### Strengths and limitations

The diversity of studies included in this analysis are a strength and a limitation. We identified similar results across a broad range of settings and used appropriate linear mixed model analysis adjusting for relevant covariates (age, sex, baseline value) and controlling for site. However, this is a post-hoc hypothesis-generating analysis, with none of the included studies designed to specifically address whether the effects of assignment to atropine eye concentrations varied by iris colour and differences in assessment protocols may mean results across studies are not exactly comparable. In the analysis of MOSAIC2 data, the washout group had previously been using atropine 0.01% eye drops, thus were not a treatment-naive control group. The number of participants in MOSAIC2 was smaller than in the pooled analysis of atropine 0.01%, which will have reduced our power to detect effects relating to atropine 0.05% use. Similarly, mesopic and photopic pupil diameter was only measured in the MOSAIC study, limiting sample size for this analysis. Given the limited number of participants using atropine 0.05% eye drops in MOSAIC2 (*n* = 66, 12 had brown iris colour), further investigation of the potential differences in atropine 0.05% efficacy with iris colour is needed.

## Conclusion

Iris colour had no clinically significant modifying effect on changes in pupil diameter or accommodative amplitude with use of 0.01% and 0.05% atropine eye drops. It is important to note that rates of treatment-related symptoms may still differ between different iris colour groups, but our results suggest that physiological changes in pupil and accommodative function are similar. This is important given it is frequently assumed that change in pupil diameter will be larger in lighter iris colours. The results of this exploratory analysis suggest that, compared with placebo, atropine 0.01% has a larger effect on inhibition of axial eye growth and myopia progression in participants with blue or green/hazel iris colours vs brown iris colours, while we did not detect a difference with atropine 0.05% treatment. This is a potentially a key consideration for clinicians prescribing atropine 0.01%; however, prospective, randomised clinical trials of 0.01% and higher-concentration atropine eye drops (e.g., 0.05%), stratified by iris colour and with appropriate controls are needed to verify this finding.

## Summary

### What was known before

- Light iris colours (e.g., blue) are known to respond differently to a single dose of anti-muscarinic eye drops, but there is little empirical evidence on eye colour differences with long-term low-concentration atropine eye drops for treating myopia progression.

### What this study adds

- This exploratory, post-hoc meta-analysis showed that there were minimal differences in change in pupil diameter or accommodative amplitude between blue/green and brown iris colours with atropine 0.01% or 0.05% eye drops.
- Children with blue/green irides showed less myopia progression relative to placebo with atropine 0.01% eye drops, but further verification is needed.

## Supplementary information

Supplementary Figure 1

Supplementary Table 1

Supplementary Table 2

Supplementary Table 3

Supplementary Table 4

## Data Availability

Data from the MOSAIC and WA-ATOM studies are available upon request to the Principal Investigators. MTS1 data are available at https://public.jaeb.org/pedig/stdy. The source of the MTS1 data is A Randomised Trial of Low-Dose Atropine for the Treatment of Myopia: Myopia Treatment Study 1. The study was funded by the National Eye Institute of the National Institutes of Health Grants EY11751, EY18810 and EY23198; and was conducted by the Pediatric Eye Disease Investigator Group (PEDIG). The analyses, content and conclusions presented herein are solely the responsibility of the authors and have not been reviewed or approved by the PEDIG group, the NEI, or the NIH.
