# Supplementary material for: Low-concentration atropine for management of myopia progression: does iris colour matter?
Source: Eye (Lond). 2026 Apr 27;40(10):1499–506. doi: 10.1038/s41433-026-04478-1 (PMC13342569; doi:10.1038/s41433-026-04478-1)
Supplement: Supplementary file 1 — Supplementary Figure 1 [file 41433_2026_4478_MOESM1_ESM.docx]

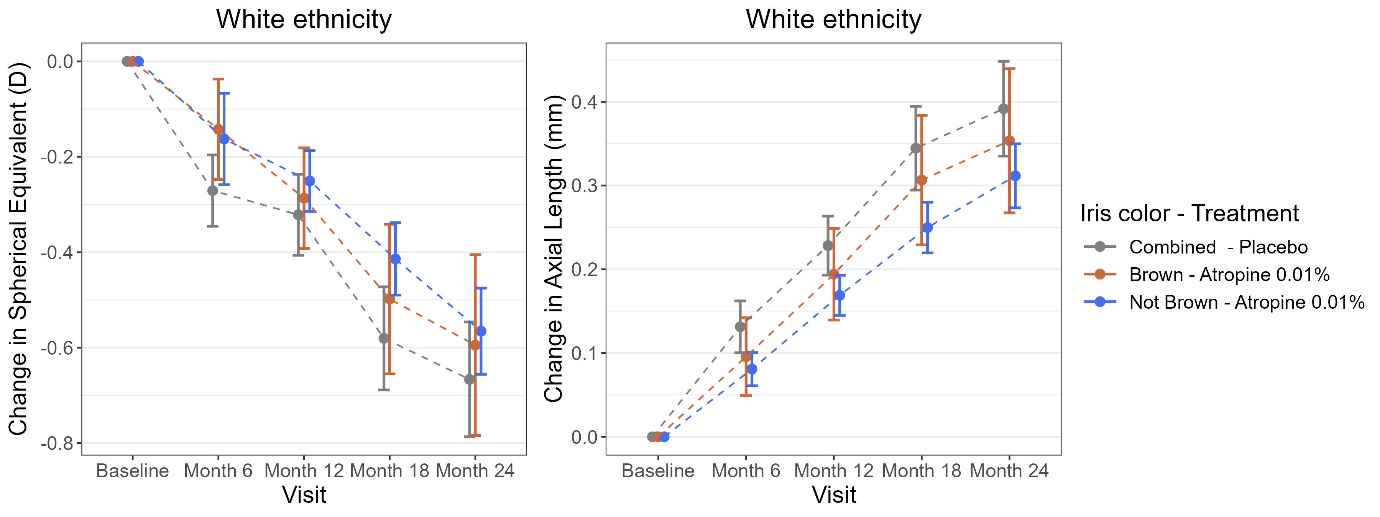


**Supplementary Figure 1:** Sensitivity analysis showing raw mean change and 95% confidence intervals for spherical equivalent and axial length outcomes in a sensitivity dataset of MOSAIC, WA-ATOM and MTS1 participants of parent-reported White ethnicity. Interactions between iris colour/treatment group and visit were significant for axial length (p=0.04), but not spherical equivalent (p=0.10).
