# Supplementary material for: Low-concentration atropine for management of myopia progression: does iris colour matter?
Source: Eye (Lond). 2026 Apr 27;40(10):1499–506. doi: 10.1038/s41433-026-04478-1 (PMC13342569; doi:10.1038/s41433-026-04478-1)
Supplement: Supplementary file 2 — Supplementary Table 1 [file 41433_2026_4478_MOESM2_ESM.docx]

**Supplementary Table 1**: Key eligibility criteria and assessment methodologies in the MOSAIC, WA-ATOM and MTS1 studies.

|  | **MOSAIC** | **WA-ATOM** | **MTS1** |
| --- | --- | --- | --- |
| **Key eligibility criteria** | |  |  |
| Age | 6-16 years | 6-16 years | 5-12 years |
| SER | ≤-1.00 D | ≤-1.50 D | -1.00 to -6.00 D |
| Astigmatism | ≤2.50 D | ≤1.50 D | ≤1.50 D |
| **Key assessments** |  |  |  |
| Assessment frequency | 6-monthly* | 6-monthly | 6-monthly |
| Treatment duration | MOSAIC1: 24 months  MOSAIC2: 12 months | 24 months | 24 months |
| Study duration | 36 months | 36 months | 30 months |
| Iris color | Examiner-assessed [blue, green, brown] | Examiner-assessed [blue, green, brown] | Parent-reported [brown, not brown] |
| Spherical equivalent refraction | Cycloplegic autorefraction | Cycloplegic autorefraction | Cycloplegic autorefraction |
| Axial length | PCI | PCI | PCI or OLCR |
| Pupillometry | PLR (Mes); Static pupil diameter (Pho, Mes) | PLR (Sco) | N/A |
| Accommodative amplitude | RAF rule, binocular and monocular | RAF rule, binocular | Near-point rule, binocular |

PCI: Partial coherence interferometry; OCT: Optical coherence tomography; OLCR: optical low coherence reflectometry; PLR: Pupil light response; Sco: Scotopic lighting conditions; Mes: Mesopic lighting conditions; Pho: Photopic lighting conditions; RAF Royal Air Force. MOSAIC1 refers to period from baseline to 24 months (nightly placebo vs atropine 0.01%) whereas MOSAIC2 refers to period from month 24 to month 36 (placebo vs tapered atropine 0.01% vs nightly atropine 0.05%).

*Month-6 visit abandoned due to the COVID-19 pandemic
