# Supplementary material for: Low-concentration atropine for management of myopia progression: does iris colour matter?
Source: Eye (Lond). 2026 Apr 27;40(10):1499–506. doi: 10.1038/s41433-026-04478-1 (PMC13342569; doi:10.1038/s41433-026-04478-1)
Supplement: Supplementary file 3 — Supplementary Table 2 [file 41433_2026_4478_MOESM3_ESM.docx]

Supplementary Table 2: Modelled interactions and pairwise differences on the effects of atropine 0.01% or 0.05% on change in accommodative amplitude in dioptres (D)

| **Accommodative amplitude (D)** | | |  |  |  |  |  |  |
| --- | --- | --- | --- | --- | --- | --- | --- | --- |
| n (not brown : brown) | Visit | Treatment | Not brown iris colour, mean (SD) | Brown iris colour, mean (SD) | Group difference | p | 3-way int. | 2-way int. |
| **MOSAIC1, WA-ATOM, MTS1** | | |  |  |  |  | 0.002 | N/A |
| 82:129 | Month 6 | Atropine 0.01% | -1.25 (4.98) | -0.51 (5.53) | -0.87 (-1.79, 0.05) | 0.06 |  |  |
| 37:55 |  | Placebo | 0.64 (3.98) | -0.11 (4.99) | 0.10 (-1.26, 1.46) | 0.89 |  |  |
| 72:115 | Month 12 | Atropine 0.01% | -0.95 (5.55) | -1.11 (5.48) | -1.07 (-2.02, -0.12) | 0.03 |  |  |
| 36:53 |  | Placebo | 0.56 (4.93) | 0.55 (5.3) | -0.50 (-1.87, 0.87) | 0.47 |  |  |
| 182:145 | Month 18 | Atropine 0.01% | -0.37 (4.71) | -0.75 (5.58) | -0.12 (-0.94, 0.70) | 0.78 |  |  |
| 91:61 |  | Placebo | -0.43 (3.27) | -0.16 (4.98) | -1.16 (-2.36, 0.04) | 0.06 |  |  |
| 182:166 | Month 24 | Atropine 0.01% | -0.94 (5.07) | -1.29 (5.75) | 0.13 (-0.68, 0.94) | 0.76 |  |  |
| 91:72 |  | Placebo | -0.88 (4.10) | -0.05 (5.64) | -1.33 (-2.50, -0.16) | 0.03 |  |  |
| **MOSAIC2** |  |  |  |  |  |  | 0.53 | 0.57 |
| 49:11 | Month 30 | Atropine 0.05% | -0.77 (4.66) | -1.18 (3.72) | 0.51 (-1.71, 2.74) | 0.65 |  |  |
| 37:15 |  | Washout | -0.35 (2.72) | -0.63 (3.53) | -0.21 (-2.16, 1.74) | 0.83 |  |  |
| 50:11 | Month 36 | Atropine 0.05% | -1.01 (4.57) | -1.82 (2.08) | 0.13 (-2.10, 2.35) | 0.91 |  |  |
| 38:16 |  | Washout | -1.32 (3.99) | -1.00 (4.77) | -0.60 (-2.53, 1.34) | 0.55 |  |  |

Not brown and Brown iris colour group columns are the raw, mean and standard deviation (SD) change from baseline. Interaction terms were used to test whether the effect of treatment varied significantly by iris colour group and visit (3-way int.) or whether the effect of treatment varied significantly with iris colour regardless of visit (2-way int.). Adjusted group difference and 95% confidence intervals are calculated using estimated marginal means (emmeans) package from a linear mixed model adjusted for age, sex and baseline value of the outcome and random intercepts for participant ID within study.
