# Supplementary material for: Low-concentration atropine for management of myopia progression: does iris colour matter?
Source: Eye (Lond). 2026 Apr 27;40(10):1499–506. doi: 10.1038/s41433-026-04478-1 (PMC13342569; doi:10.1038/s41433-026-04478-1)
Supplement: Supplementary file 5 — Supplementary Table 4 [file 41433_2026_4478_MOESM5_ESM.docx]

Supplementary Table 4: Mean changes and modelled interactions and pairwise differences on the effects of atropine 0.01% or 0.05% vs placebo by iris colour group on change in spherical equivalent refraction in dioptres and axial length in millimetres.

| **Spherical equivalent refraction (D)** | | | | | | | | |
| --- | --- | --- | --- | --- | --- | --- | --- | --- |
| n (atropine:  placebo) | Visit | Iris colour | Atropine 0.01% | Placebo | Adjusted group difference (95% CI) | p | 3-way int. | 2-way int. |
| **MOSAIC1, WA-ATOM, MTS1** | | |  |  |  |  | 0.04 | N/A |
| 82:37 | Month 6 | Not brown | -0.17 (0.42) | -0.27 (0.28) | 0.17 (0.04, 0.30) | 0.01 |  |  |
| 129:56 |  | Brown | -0.23 (0.38) | -0.29 (0.25) | 0.06 (-0.06, 0.19) | 0.33 |  |  |
| 185:97 | Month 12 | Not brown | -0.25 (0.43) | -0.33 (0.46) | 0.11 (0.001, 0.22) | 0.05 |  |  |
| 153:69 |  | Brown | -0.40 (0.49) | -0.42 (0.38) | 0.01 (-0.11, 0.14) | 0.85 |  |  |
| 183:90 | Month 18 | Not brown | -0.42 (0.51) | -0.60 (0.56) | 0.21 (0.10, 0.32) | <0.001 |  |  |
| 146:61 |  | Brown | -0.59 (0.63) | -0.60 (0.54) | 0.01 (-0.11, 0.14) | 0.84 |  |  |
| 182:91 | Month 24 | Not brown | -0.57 (0.61) | -0.69 (0.62) | 0.17 (0.06, 0.28) | 0.003 |  |  |
| 166:72 |  | Brown | -0.77 (0.73) | -0.67 (0.58) | -0.08 (-0.20, 0.04) | 0.21 |  |  |
| **MOSAIC2** |  |  | Atropine 0.05% | Washout |  |  | 0.67 | 0.06 |
| 49:37 | Month 30 | Not brown | -0.04 (0.29) | -0.13 (0.27) | 0.09 (-0.01, 0.20) | 0.08 |  |  |
| 11:15 |  | Brown | 0.07 (0.25) | -0.21 (0.31) | 0.30 (0.11, 0.49) | 0.002 |  |  |
| 51:38 | Month 36 | Not brown | -0.12 (0.31) | -0.23 (0.31) | 0.08 (-0.02, 0.19) | 0.12 |  |  |
| 11:16 |  | Brown | -0.02 (0.21) | -0.28 (0.39) | 0.29 (0.10, 0.48) | 0.003 |  |  |
| **Axial length (mm)** | | | | | | | | |
| n (atropine:  placebo) | Visit | Iris colour | Atropine 0.01% | Placebo | Adjusted group difference (95% CI) | p | 3-way int. | 2-way int. |
| **MOSAIC1, WA-ATOM, MTS1** | | |  |  |  |  | 0.01 | N/A |
| 82:37 | Month 6 | Not brown | 0.09 (0.09) | 0.13 (0.11) | -0.07 (-0.12, -0.02) | 0.006 |  |  |
| 128:56 |  | Brown | 0.12 (0.15) | 0.13 (0.11) | -0.01 (-0.06, 0.04) | 0.56 |  |  |
| 182:97 | Month 12 | Not brown | 0.17 (0.16) | 0.23 (0.19) | -0.06 (-0.10, -0.01) | 0.008 |  |  |
| 154:69 |  | Brown | 0.23 (0.21) | 0.25 (0.20) | -0.005 (-0.05, 0.04) | 0.85 |  |  |
| 182:91 | Month 18 | Not brown | 0.23 (0.15) | 0.35 (0.26) | -0.10 (-0.14, -0.06) | <0.001 |  |  |
| 147:60 |  | Brown | 0.33 (0.27) | 0.33 (0.22) | -0.006 (-0.06, 0.04) | 0.82 |  |  |
| 182:91 | Month 24 | Not brown | 0.32 (0.26) | 0.40 (0.29) | -0.09 (-0.13, -0.05) | <0.001 |  |  |
| 167:71 |  | Brown | 0.42 (0.32) | 0.38 (0.27) | 0.03 (-0.02, 0.07) | 0.30 |  |  |
| **MOSAIC2** |  |  | Atropine 0.05% | Washout |  |  | 0.96 | 0.10 |
| 49:37 | Month 30 | Not brown | 0.05 (0.10) | 0.09 (0.13) | -0.03 (-0.08, 0.01) | 0.18 |  |  |
| 11:15 |  | Brown | 0.02 (0.08) | 0.11 (0.12) | -0.11 (-0.20, -0.03) | 0.009 |  |  |
| 51:38 | Month 36 | Not brown | 0.09 (0.15) | 0.12 (0.16) | -0.03 (-0.08, 0.02) | 0.20 |  |  |
| 11:16 |  | Brown | 0.07 (0.11) | 0.17 (0.19) | -0.11 (-0.20, -0.03) | 0.01 |  |  |

SD: standard deviation; CI: confidence interval. Atropine 0.01%, placebo, Atropine 0.05% and washout group columns are the raw, mean and standard deviation (SD) change from baseline. Interaction terms were used to test whether the effect of treatment varied significantly by iris colour group and visit (3-way int.) or whether the effect of treatment group varied significantly with iris colour regardless of visit (2-way int.). Adjusted group difference and 95% confidence intervals are calculated using estimated marginal means (emmeans) package from a linear mixed model adjusted for age, sex and baseline value of the outcome and random intercepts for participant ID within study.
